# Clinical and Laboratory Diagnostic Features of Kyasanur Forest Disease: A Study From Wayanad, South India

**DOI:** 10.7759/cureus.20194

**Published:** 2021-12-06

**Authors:** Vineeth Gladson, Hisham Moosan, Sheela Mathew, Dineesh P

**Affiliations:** 1 General Medicine, Government Medical College Hospital, Kozhikode, IND; 2 Community Medicine, DM Wayanad Institute of Medical Sciences, Wayanad, IND; 3 Infectious Diseases, Government Medical College Hospital, Kozhikode, IND; 4 Department of Health Services, Health Services, Government of Kerala, Kerala, IND

**Keywords:** haemaphysalis spinigera, hard tick, monkey fever, kfd, kyasanur forest disease

## Abstract

Background

Kyasanur forest disease (KFD), also known as monkey fever, was first recognized in the Shimoga district of Karnataka, India, in 1957. This study was conceived to address the paucity of medical literature on KFD, to describe the clinical and laboratory features of real-time reverse-transcriptase-polymerase chain reaction (rRT-PCR) confirmed cases of KFD, and to detect any change in the clinical picture and presentation of the disease over the last 30 years.

Aim

The study sought to document the clinical and laboratory features of Kyasanur forest disease (KFD), a tick-borne arboviral disease, now emerging in many parts of southern India.

Material and methods

This was a retrospective study using secondary data of patients with real-time reverse transcription-polymerase chain reaction (rRT-PCR)-confirmed KFD in a secondary care hospital in Wayanad, Kerala, India.

Results

Sixty rRT-PCR-proven KFD patients were included in the study. Commonly noted clinical symptoms were fever (98%), headache (80%), body ache (86%), vomiting (61%), and prostration (83%). Relative bradycardia, hypotension (45%), and oral lesions (23%) were the frequent physical signs. The median total leukocyte count and median platelet count at admission were 2600 per μL and 1.62 per μL, respectively. The median erythrocyte sedimentation rate was 10 mm/hr. Urinary sediments and albuminuria were seen in 66% and 60% respectively. The major complications observed were neurological complications (23%), bleeding manifestations (20%), and persistent shock (20%). The common neurological complications were seizures, altered sensorium, aseptic meningitis, and focal neurological deficits. The case fatality rate in the study was 6.7%.

Conclusions

The clinical picture of a prostrating viral syndrome in an epidemiological setting of KFD with marked leucopenia, moderate thrombocytopenia, low erythrocyte sedimentation rate (ESR), albuminuria, urinary sediments, and moderately elevated transaminases help in making an early diagnosis of KFD. Neurological complications in the initial two weeks are associated with poor outcomes.

## Introduction

Kyasanur forest disease (KFD), also known as monkey fever, was first recognized in the Shimoga district of Karnataka, India, in 1957 [1]. It is caused by the Kyasanur forest disease virus (KFDV), a member of the tick-borne virus of the family Flaviviridae [2]. The Alkhurma virus of Saudi Arabia is considered to be a variant of the Kyasanur forest disease virus [3]. Hard tick (Hemaphysalis spinigera) is the reservoir and vector of the KFDV and once infected, it remains infected for life. Monkeys and rodents are the natural hosts for KFDV. Monkeys and humans develop clinically symptomatic KFD, of which humans are accidental dead-end hosts [4]. The incubation period of KFD is documented to be between three and eight days following an infected tick bite [5].

The outbreaks of KFD occur seasonally. It begins in the month of December, peaks between January and April, and declines by June [1,4]. Monkey deaths occurring in the forests and nearby communities are considered as a harbinger of an outbreak. Animal grazing, forest visits, monkey deaths, and collection of leaf heaps near houses are the major risk factors associated with the disease [6]. The first symptoms of KFD include fever, myalgia, headache, prostration, vomiting, and diarrhea.

After its discovery in 1957, KFD was confined to five districts (Shimoga, Chikkamangalore, Uttara Kannada, Dakshina Kannada, and Udupi) of the state of Karnataka in Southern India for the last five decades [3]. But in 2012, KFDV was isolated from the monkey specimens in Nilgiris in the state of Tamil Nadu and human cases were reported from Chamarajnagar district of Karnataka [3,7]. In 2013, the first case of KFD in the state of Kerala was reported in Wayanad district, which neighbors the Chamarajnagar district of Karnataka.

In 2015, a major epidemic of KFD occurred in the Wayanad district of Kerala, with 102 confirmed patients [3]. The outbreak started among a group of women who were working in the forest. Although initially the outbreak was seen mainly among the tribal communities who dwell in and around the forest, later it spread to involve the general population as well. Further outbreaks of KFD were reported from the state of Goa in the year 2015 and Maharashtra in the years 2016 and 2017 [8-9]. In the last five years, KFD is slowly emerging as a life-threatening infection in many new states adjacent to the Western Ghats [10]. Currently, the original articles regarding the clinical and laboratory profile of KFD are limited to the initial case reports by Work et al. in Webb et al. and the landmark study by Adhikari Prabha et al. conducted in 1984-85, which had used clinical and serology criteria to define cases of KFD [1,11-12]. The study was conducted to describe the clinical and laboratory features of real-time reverse-transcriptase-polymerase chain reaction (rRT-PCR)-confirmed cases of KFD. As such, there is a paucity of medical literature on rRT-PCR-proven cases of KFD, and we also wanted to detect any change in the clinical picture and presentation of the disease over the last 30 years.

## Materials and methods

The study was a retrospective observational study. In January 2015, a group of laborers who went for work in the Kurichiad forest of Wayanad district of Kerala were affected by high-grade fever and got admitted to a local hospital [13]. These patients were later diagnosed as KFD virus-positive, from Manipal center for virus research, Manipal Academy of Higher Education (designated KFD testing laboratory). The diagnosis was made using the rRT-PCR assay targeting NS 5 gene on the blood specimen. Further epidemics of KFD were observed for the next five years but only sporadic cases were present. Data were collected after obtaining ethical clearance, from positive cases both from the epidemic cluster and further observed sporadic cases. The last case observed was in February 2021. The selection criteria were rRT-PCR positivity of NS 5 gene of KFD virus.

The data included demographic, clinical, and laboratory parameters. Age, sex, the month of admission, locality, and presence of comorbidities like hypertension, diabetes, chronic obstructive pulmonary disease, and coronary artery disease were noted for all patients. History of fever, myalgia, arthralgia, headache, backache, gastrointestinal tract symptoms, respiratory symptoms, and nervous system symptoms were entered. Details of general examination like pulse rate, respiratory rate, blood pressure, pallor, lymph node enlargement, edema, evidence of arthritis, skin rash, conjunctival congestion, oral ulcers, and system examination findings were recorded. Laboratory data included hematological and biochemical investigations. Whenever additional tests like ECG, chest X-ray, echocardiography, cerebrospinal fluid (CSF) study, and imaging of the brain were performed, they were also entered. A pulse rate of more than 100 beats per minute was taken as tachycardia, respiratory rate of more than 20 breaths per minute as tachypnea, and systolic blood pressure < 90 mmHg as hypotension. Data were entered in an Excel sheet (Microsoft Corporation, Redmond, WA) and analyzed in EpiData Analysis V2.2.3.187 (EpiData Association, Denmark).

## Results

The total number of patients with rRT-PCR-confirmed KFD and were included in this study was 60. The mean (SD) age of the patients in the study group was 38 (13.5) years. There was considerable female predominance among the sample (73%). The majority of patients belonged to the tribal communities. Common symptoms and signs are shown in Table 1.

**Table 1:** Clinical manifestations of Kyasanur forest disease patients

Clinical Manifestation	Yes (%)	No (%)
Fever	59 (98.3)	1 (1.7)
Headache	48 (80.0)	12 (20.0)
Body ache	52 (86.7)	8 (13.3)
Vomiting	37 (61.7)	23 (38.3)
Loose stools	23 (38.3)	37 (61.7)
Prostration	50 (83.3)	10 (16.7)
Bleeding	12 (20.0)	48 (80.0)
Cough	17 (28.3)	43 (71.7)
Throat pain	12 (20.0)	48 (80.0)
Loss of appetite	48 (80.0)	12 (20.0)
Breathlessness	3 (5.0)	57 (95.0)
Seizures	5 (8.3)	55 (91.7)
Altered sensorium	9 (15.0)	51 (85.0)
Abdominal pain	18 (30.0)	42 (70.0)
Conjunctival congestion	4 (6.7)	56 (93.3)
Lymphadenopathy	5 (8.3)	55 (91.7)
Oral ulcers	14 (23.3)	46 (76.7)
Hepatomegaly	10 (16.7)	50 (83.3)
Splenomegaly	2 (3.3)	58 (96.7)
Neck stiffness	3 (5.0)	57 (95.0)
Vital signs	Mean	Standard deviation
Pulse (beats per minute)	77.6	11.2
Systolic blood pressure, mm of Hg	97.53	16.81
Diastolic blood pressure, mm of Hg	65.50	11.99

The median of total leukocyte count on presentation was 2600 per μL (Table 2).

**Table 2:** Laboratory parameters of patients with Kyasanur forest disease * Mean (SD), #Median (IQR) ESR: erythrocyte sedimentation rate

Lab Parameter	Values
Hemoglobin, g/dL*	10.56 (2.19)
Total leukocyte count at presentation, per μL^#^	2600 (2000-4300)
Lowest total leukocyte count, per μL^#^	1950 (1325-2800)
Neutrophil count, % *	63.22(11.16)
Platelet count at presentation, x10^5 ^per μL ^#^	1.62(1.09-2.00)
Lowest Platelet count, x10^5 ^per μL ^#^	1.10(0.73-1.67)
ESR, mm/hr^#^	10.00 (5.2-15.75)
Total bilirubin, mg/dL^ #^	0.9(0.8-0.9)
Aspartate aminotransferase, U/L^#^	126.50(46.25-268.75)
Alanine aminotransferase, U/L^#^	80(36-186)
Alkaline phosphatase, U/L^#^	130(90.00-211.50)
Creatinine, mg/dL*	0.92(1.31)
Urine analysis	n (%)
Urine sediments	40(66.7)
Albumin	36(60)

The lowest total leukocyte count at presentation was 800 per μL. The mean of the lowest total leukocyte count during hospital stay was 2600 per μL and the median was 1950 per μL. The median of lowest platelet count during hospital stay was 110,000 per μL. The lowest platelet count noted was 32,000 per μL. The median ESR was 10 mm/hr. Urinary sediments and albuminuria were seen in 66.7% (n=40) and 60% (n=36), respectively. The median values for AST and ALT in the study were 126.50 and 80 IU/L, respectively. Three patients had AST values above 1000 IU/L and one patient had an ALT value above 1000 IU/L. The major comorbidities were anemia (n=11), diabetes (n=4), hypertension (n=1), and alcoholism (n=2).

The predictors of mortality were identified using univariate analysis. Breathlessness, presence of concurrent conditions like anemia, diabetes, hypertension, and alcoholism and neurological manifestations like neck stiffness (p < 0·0001), altered sensorium (p<0·001), and seizures (p<0·001) were found to be significantly associated with mortality. The difference in the age of survivors and non-survivors was significant (p < 0·03). The mean pulse rate, systolic blood pressure, and diastolic blood pressure at the time of presentation at the health care facility were significantly different between survivors and non-survivors (Table 3).

**Table 3:** Comparison of clinical manifestations between survivors and non-survivors of Kyasanur forest disease

Clinical Manifestation	Non-Survivors	Survivors	p-value
Fever	3(5.1)	56(94.9)	0.06
Headache	2(4.2)	46(95.8)	0.17
Body ache	2(3.8)	50(96.2)	0.08
Vomiting	2(5.4)	35(94.6)	0.63
Loose stools	2(8.7)	21(91.3)	0.63
Prostration	4(8)	46(92.0)	1.00
Cough	1(5.9)	16(94.1)	1.00
Throat pain	0(0.0)	12(100)	0.57
Loss of appetite	4(8.3)	44(91.7)	0.57
Breathlessness	3(100)	0(0)	<0.01
Seizures	4(80.0)	1(20.1)	<0.001
Altered sensorium	4(44.4)	5(55.6)	<0.001
Bleeding manifestations	1(8.3)	11(91.7)	1.00
Abdominal pain	1(5.6)	17(94.4)	1.00
Concurrent condition(s)	4(21.1)	15(78.9)	<0.01
Conjunctival congestion	1(25.0)	3(75.0)	0.24
Lymphadenopathy	0(0.0)	5(100)	1.00
Oral ulcers	2(14.3)	12(85.7)	0.22
Hepatomegaly	1(10.0)	9(90.0)	0.57
Splenomegaly	0(0.0)	2(100)	1.00
Neck stiffness	3(100)	0(0.0)	< .0001>
Vital signs	Non-Survivors	Survivors	p Value
Pulse (beats per minute)	65.50(16.84)	78.50(10.42)	0.02
Systolic blood pressure, mm of Hg	80.0(20.00)	98.79(160.4)	0.02
Diastolic blood pressure, mm of Hg	50.00(27.08)	66.61(9.78)	<0.01

The difference in the median of aspartate transaminase (AST), platelet count, and serum creatinine was also found to be significant between survivors and non-survivors (Table 4).

**Table 4:** Comparison of laboratory parameters between survivors and non-survivors of Kyasanur forest disease * Mean (SD), #Median (IQR) ESR: erythrocyte sedimentation rate

Lab Parameters	Non-Survivors (SD / IQR)	Survivors (SD / IQR)	p-value
Hemoglobin, g/dL*	11.53(2.91)	10.49(2.14)	0.36
Total leukocyte count, per μL^#^	2100(1200-7800)	2600(2000-4300)	0.47
Lowest total leukocyte count, per μL^#^	2100(1000-7800)	1850(1325-2800)	0.71
Neutrophil count, % *	55(48-62)	64(58-72.75)	0.08
Platelet count at presentation, x10^5 ^per μL ^#^	0.85(0.50-1.70)	1.66(1.10-2.15)	0.03
Lowest platelet count, x10^5 ^per μL ^#^	0.85(0.50-1.70)	1.10(0.76-1.67)	0.36
ESR, mm/hr^#^	10(5-20)	10(6-15.75)	0.79
Aspartate aminotransferase, U/L^#^	315(200-610)	116.50(43.75-249.00)	0.02
Alanine aminotransferase, U/L^#^	166(80-381)	63(36-180)	0.16
Alkaline phosphatase, U/L^#^	231(100-340)	128(90-185.25)	0.15
Creatinine, mg/dL*	1.50(0.47)	0.88(0.25)	<0.001
Urinary sediments n(%)	3(7.5)	37(92.5)	1.00
Urine albumin n(%)	4(11.1)	32(88.9)	0.14

There was no significant difference in the bleeding manifestations between survivors and non-survivors.

Bleeding manifestations, persistent shock, neurological complications, hepatitis, and renal dysfunction were the common complications observed in our study (Table 5).

**Table 5:** Complications observed in patients with Kyasanur forest disease

Complications	n	%
Neurological complications	14	23.3
Altered sensorium	9	15.0
Aseptic meningitis	3	5.0
Focal neurological deficits	4	6.7
Seizures	5	8.3
Hemorrhagic manifestations	12	20.0
Persistent shock (SBP < 90 mm of Hg )	12	20.0
Hepatitis (Bilirubin >1.3 mg/dL or AST/ALT > 400 U/L)	10	16.7
Acute kidney injury (Creatinine >1.5 mg/dL)	6	10.0
Myocarditis	2	3.3
Hemolysis	2	3.3
Pneumonitis	2	3.3
Death	4	6.7

Menorrhagia, epistaxis, melaena, hemoptysis, and bleeding from the mouth were the common bleeding manifestations observed. Seizures, altered sensorium, aseptic meningitis/encephalitis as evidenced by CSF findings, facial palsy, and spastic paraparesis were the neurological complications. Facial palsy, spastic paraparesis, and aseptic meningitis were documented after the third week. Four patients succumbed to death in the second week of illness. All four patients had multiple episodes of seizures and one patient had refractory hypotension. Hepatitis was defined as serum bilirubin > 1.3 mg/dL or AST/ALT > 400 U/L, and renal dysfunction was defined as creatinine > 1.5 mg/dL. One patient with persistent cytopenia had evidence of hemophagocytic lymphohistiocytosis (HLH).

## Discussion

KFD was initially reported by Work et al in 1957 [1]. The only descriptive study regarding the clinical profile of patients with KFD was conducted by Adhikari et al. in the year 1984-85 [12]. Three decades after this seminal study, we are reporting the clinical and laboratory profile of 60 rRT-PCR-proven KFD patients.

As in most undifferentiated febrile illnesses, the common symptoms documented at the time of presentation were fever, headache, myalgia, vomiting, and prostration. But the severity of prostration (83·3%) was much more than most of the other fevers. Loose stools were documented in 23 patients (38·3%), and a minority had an acute diarrheal disease like presentation with watery loose stools and vomiting. Throat pain was reported in some patients, which was occasionally associated with vesicular lesions in the oral cavity, oral ulcers, and congestion of pharyngeal mucosa. Pavri et al. had mentioned similar findings in their study [14]. Bleeding manifestations documented were mostly mild and improved with symptomatic management.

The other common signs observed were oral ulcers in 23% and hepatomegaly in 17%. Other studies have reported glossitis and stomatitis in 11% of the patients and hepatomegaly in 50% of the patients [12]. But the high rates of conjunctival congestion reported in previous studies were not seen in our study. The low percentage of conjunctival congestion in our study compared to the previous studies may be because only severe bulbar conjunctival congestion was recorded.

There was no tachycardia and the pulse rate was on the lower side despite the patients having fever and hypotension. Hypotension, defined as systolic blood pressure ≤ 90 mm of Hg at presentation, was reported in 45% of the patients. It was observed in 61% of the patients in other studies [12]. Though myocardial involvement was suspected as the cause for hypotension, elevated cardiac biomarkers could not be documented in most patients with persistent hypotension.

The total leukocyte count on presentation was less than 4000 per μL in 69% of the patients and subsequently, the counts dropped to less than 4000 per μL in 80% of the patients (Table 2). The median total leukocyte count on presentation was 2600 per μL (Table 2). The lowest total leukocyte count during hospitalization was less than 2800 per μL in 75% of the patients. This level of significant leucopenia was helpful in early clinical suspicion of KFD. The thrombocytopenia was only moderate. Unlike dengue fever, the leucopenia was more profound than the thrombocytopenia. Though low total count was a sensitive parameter in suspecting KFD, it was not found to be a predictor of mortality on further analysis (Table 4). One symptomatic patient with a rapid drop of all three lineages was subjected to a bone marrow study, which was suggestive of hemophagocytosis. Serum ferritin and triglyceride were high and ESR drop was also demonstrated, suggesting a diagnosis of HLH. 

The presence of urinary sediments and albuminuria was documented in more than 60% of the patients in this study. Work et al. had mentioned the presence of granular casts and red cells in the urine in his initial reports, but albuminuria has not been reported by any prior investigator [1]. Most patients had elevated levels of liver enzymes with normal bilirubin and creatinine. As in most arboviral illnesses, the elevation in AST was greater than the elevation in ALT. In an epidemiological setup of KFD, if a patient presents with fever and severe prostration, physical examination showing bradycardia and hypotension, and laboratory parameters showing cytopenia, low ESR, and elevated transaminases (AST>ALT), the clinical probability of KFD is high.

The neurological manifestations noted in the initial two weeks were seizures, altered sensorium, and neck stiffness, all of which carried a grave prognosis. All mortality in our study occurred in the first two weeks of presentation. The case fatality rate (6.7%) in our study was much lower than the study by Adhikari et al., which had a 33% case fatality rate [12] but our study was comparable to the study by Work et al. [1]. The neurological complications noted during the biphasic phase (third and fourth weeks) were aseptic meningitis, encephalitis, upper motor neuron facial palsy, and upper motor neuron paraparesis. Two patients presented in the biphasic phase with spastic paraparesis and severe ataxia, whose MRI was normal, were treated with steroids, and had a good recovery. CSF was analyzed in six patients who had neurological manifestations. The CSF study was normal in three patients; two patients showed lymphocytic pleocytosis and one patient showed increased protein content. CSF pleocytosis and other abnormalities, mostly in the biphasic phase, have been reported by other studies and our study concurs with these findings [11-12]. The late neurological complications noted were ataxia, tremors, headaches, neuropsychiatric manifestations, visual disturbances, and deafness.

In the initial reports of KFD, hemorrhagic features of the disease were emphasized and no neurological manifestations were reported [1]. Webb et al. observed neurological findings like headache, neck stiffness, mental disturbance, tremors, and CSF pleocytosis [11]. Adhikari et al. noted hemorrhagic symptoms in 54 out of 100 cases of KFD and altered sensorium in 45 patients. Twenty-four patients had generalized convulsions that were associated with a grave prognosis [12]. Basu A et al. described the histopathology and immunohistochemistry findings in experimentally infected infant CD-1 mice with an early passage human KFD virus isolate. The array of changes included gliosis, inflammatory response, necrosis, neural loss, and syncytium formation in the mid and hindbrain structures [15]. Sawatsky et al. conducted a study to compare the pathogenesis of KFDV and Alkhurma hemorrhagic fever virus (AHFV) in a mouse model. KFDV was seen replicating primarily in the brain and to a lesser extent in the lung. The virus could not be detected in the liver, spleen, or plasma, whereas high titers of AHFV were detected in the visceral organs (kidney, spleen, and liver) but not in the brain [16]. In the light of these experimental findings and the observations from our study, we would like to suggest that KFD may be better understood as a viral syndrome with both neurological and hemorrhagic complications. The neurological complications were associated with a poor outcome in our study.

Most patients were managed conservatively without antibiotics. Patients with hypotension were managed with intravenous fluids and non-responders were given inotropes. Convalescence was prolonged and many patients had lethargy months after presentation.

Limitations and strengths

The study relied on estimates from a discrete but small group of patients. The study sample may not have been representative of the whole spectrum of complications, as some critically ill patients were managed initially in tertiary care centers.

Only NS 5 gene rRT-PCR-positive patients (rRT-PCR is the gold standard for the diagnosis of KFD) were included in the study. This is the first study to describe the clinical, hematological, and biochemical profile of rRT-PCR-confirmed cases of Kyasanur forest disease. As the hospital was in the epicenter of the outbreak, even milder variants of the disease were diagnosed and included in the study.

## Conclusions

The clinical picture of a prostrating viral syndrome in an epidemiological setting of KFD with marked leucopenia, moderate thrombocytopenia, low ESR, moderately elevated transaminases, urinary sediments, and albuminuria help in making an early diagnosis of KFD in most patients. A low total leukocyte count in a patient with a clinical-laboratory picture of viral fever should trigger suspicion for KFD. Neurological complications in the first two weeks were associated with a poor prognosis.
